# Role of *SSD1* in Phenotypic Variation of *Saccharomyces cerevisiae* Strains Lacking *DEG1*-Dependent Pseudouridylation

**DOI:** 10.3390/ijms22168753

**Published:** 2021-08-15

**Authors:** Bahar Khonsari, Roland Klassen, Raffael Schaffrath

**Affiliations:** Institut für Biologie, Fachgebiet Mikrobiologie, Universität Kassel, Heinrich-Plett-Str. 40, D-34132 Kassel, Germany; bahar.khonsari@gmail.com

**Keywords:** yeast, *SSD1*, pseudouridine, tRNA modification

## Abstract

Yeast phenotypes associated with the lack of wobble uridine (U_34_) modifications in tRNA were shown to be modulated by an allelic variation of *SSD1*, a gene encoding an mRNA-binding protein. We demonstrate that phenotypes caused by the loss of Deg1-dependent tRNA pseudouridylation are similarly affected by *SSD1* allelic status. Temperature sensitivity and protein aggregation are elevated in *deg1* mutants and further increased in the presence of the *ssd1-d* allele, which encodes a truncated form of Ssd1. In addition, chronological lifespan is reduced in a *deg1 ssd1-d* mutant, and the negative genetic interactions of the U_34_ modifier genes *ELP3* and *URM1* with *DEG1* are aggravated by *ssd1-d*. A loss of function mutation in *SSD1*, *ELP3*, and *DEG1* induces pleiotropic and overlapping phenotypes, including sensitivity against target of rapamycin (TOR) inhibitor drug and cell wall stress by calcofluor white. Additivity in *ssd1 deg1* double mutant phenotypes suggests independent roles of Ssd1 and tRNA modifications in TOR signaling and cell wall integrity. However, other tRNA modification defects cause growth and drug sensitivity phenotypes, which are not further intensified in tandem with *ssd1-d*. Thus, we observed a modification-specific rather than general effect of *SSD1* status on phenotypic variation in tRNA modification mutants. Our results highlight how the cellular consequences of tRNA modification loss can be influenced by protein targeting specific mRNAs.

## 1. Introduction

Post-transcriptional RNA modifications are abundant in tRNA, where they may support stability, integrity, and translational efficiency [1,2,3]. Different modifications are introduced at different positions of specific tRNAs. Some modifications are installed in a sequential order, and several modification genes show strong genetic interactions because independent modifications may contribute to the same tRNA functional aspect [4,5]. Extensive functional redundancy may partly explain why no loss-of-function phenotypes are observable for many of the conserved modification genes. However, some specific modification genes are linked to growth phenotypes in the yeasts *Saccharomyces cerevisiae* and *Schizosaccharomyces pombe* [6]. An tRNA modification that is important for the normal growth of yeast cells is pseudouridylation at positions 38 and 39 (Ψ_38/39_), which is introduced by pseudouridine synthase Deg1. tRNA^Gln^_UUG_ overexpression suppresses the general growth defects of *deg1* mutants, revealing a functional dependency of this tRNA on Deg1-dependent Ψ_38__/39_ [7,8]. Pseudouridylation is a frequent tRNA modification that can be found in all parts of tRNA [9,10,11,12]. There are several additional Ψ synthases responsible for pseudouridylation at other tRNA positions, and defects in some of these enzymes (including the Deg1 orthologue Pus3) are linked to neurodegenerative diseases such as intellectual disability in humans [13,14]. In yeast, the absence of Deg1 also influences neutral lipid content, amino acid levels, and sensitivity against rapamycin (an inhibitor of the TORC1 (target of rapamycin) kinase complex [15,16,17,18]), which might contribute to the general growth defect.

Yeast *DEG1* exhibits strong negative genetic interactions with various genes involved in the formation of 5-methoxycarbonylmethyl-2-thiouridine (mcm⁵s^2^U_34_). The latter tRNA modification is found in the wobble positions of tRNA^Gln^_UUG_, tRNA^Glu^_UUC_, and tRNA^Lys^_UUU_, and two separate pathways are required for its synthesis [19,20,21]. The Elongator complex (composed of proteins Elp1-Elp6) and additional accessory proteins introduce the methoxycarbonylmethyl (mcm^5^) residue at the C5 position of the wobble uridine (U_34_) [22]. An independent pathway involving Uba4, Urm1, and Ncs2/Ncs6 is responsible for U_34_ thiolation at C2, completing the mcm^5^s^2^U_34_ composite [19,20,23]. Combining defects in either U_34_ thiolation or C5 modification with a *deg1* mutation results in strong synthetic growth and/or temperature sensitivities that are, in part, suppressible by tRNA^Gln^_UUG_ overexpression [7,8]. This points to a functionally relevant collaboration of the anticodon loop modifications at U_34_ and U_38_ in tRNA^Gln^_UUG_. Similar functional collaborations between modified U_34_ and other anticodon loop modifications have been demonstrated [7,24,25]. A strong negative genetic interaction occurs even among the genes involved in the C2 and C5 modifications of U_34_ itself [26,27,28]. Several of the combined anticodon loop modification defects result in synthetic temperature sensitivities occurring in tandem with an accumulation of protein aggregates, which may form in response to ribosome slow-down at the individual hard-to-translate codons [7,28,29]. It is assumed that the protein aggregate induction contributes to the growth and temperature sensitivity phenotypes of the combined modification mutants.

Interestingly, the strength of double mutant phenotypes for the C2 and C5 hypomodification of U_34_ is modulatable by the allelic variant of a gene (*SSD1*) encoding an mRNA-binding protein [30]. Ssd1 binds mRNAs encoding cell wall proteins and represses their translation under stress conditions [31,32]. The latter involves the association of Ssd1 and bound mRNA with cytoplasmic processing bodies (P-bodies) and requires the prion-like protein domain of Ssd1 [33]. Under favorable growth conditions, Ssd1 is thought to mediate the delivery of cell wall protein-encoding mRNA to sites of polarized growth [33]. Common laboratory yeast strains such as S288C and W303-1B [34] carry the allelic variants termed *SSD1-v* or *ssd1-d,* the latter of which encodes a truncated defective Ssd1 form. *SSD1-v* (suppressor of *SIT4* deletion) suppresses the lethal effect of *SIT4* phosphatase gene deletion [35], while *ssd1-d* cannot but shortens chronological lifespan [36] and enhances the phenotypes of Elongator mutants [30], including a more pronounced genetic interaction of *ELP3* and the thiolase gene *NCS2,* as well as the enhanced accumulation of protein aggregates in the combined *elp3 ncs2* mutant [30]. It remained unknown, however, whether phenotypic modulation by *SSD1* status is specific to Elongator-dependent U_34_ modifications or can be generalized to phenotypes from other tRNA modification mutants. In this study, we reveal a modulation of *deg1* mutant phenotypes by *SSD1* and demonstrate that other tRNA modification defects are not similarly affected. A possible relevance of independent cell wall integrity defects induced by *ssd1-d*, *elp3*, and *deg1* is discussed.

## 2. Results

### 2.1. Comparison of elp3 and deg1 Mutant Phenotypes in ssd1-d and SSD1-v Strains

**Figure 1:**
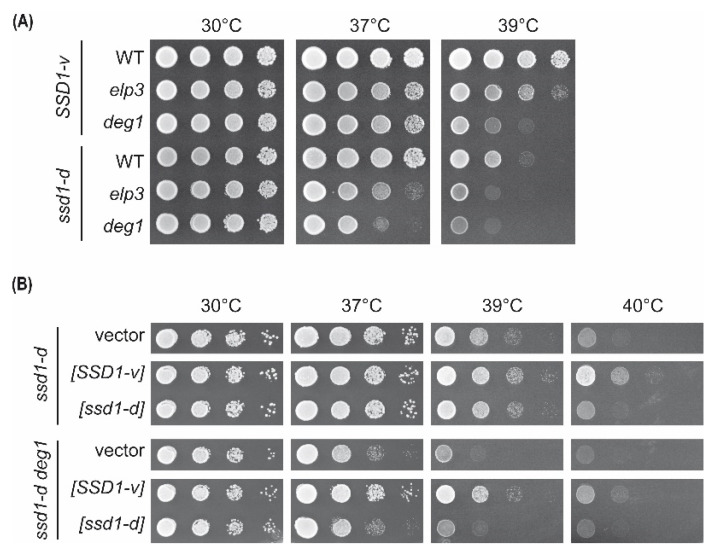
Temperature sensitivity of *elp3* and *deg1* mutants in the *SSD1-v* and *ssd1-d* strain backgrounds. (**A**) Wild type (WT) and indicated mutants were serially diluted, spotted on yeast extract–peptone–dextrose (YPD) plates, and incubated at the specified temperature for 48 h. (**B**) The WT and *deg1* mutant in the *ssd1-d* background containing an empty vector, *SSD1-v-*, or *ssd1-d*-containing plasmids were serially diluted, spotted on YPD plates, and incubated at the indicated temperature for 48 h.

To analyze whether the *SSD1* allelic variants influence phenotypes induced by lack of pseudouridine synthase Deg1 in the yeast *S. cerevisiae*, we generated *deg1* mutants in both *SSD1-v* (BY4741) and *ssd1-d* (W303-1B) strain backgrounds. For comparison, we deleted *ELP3* in both backgrounds and analyzed the growth of all strains in response to elevated cultivation temperatures. In both cases, the modification defects were found to cause a fitness defect at elevated temperatures (Figure 1A).

As previously observed, the growth defect of *deg1* mutants was more pronounced than that of *elp3.* The difference was observable in both *ssd1-d* and *SSD1-v* (Figure 1A). However, in the *ssd1-d* strain, the growth defect of both tRNA modification mutants at 37 °C was enhanced compared to that with the *SSD1-v* background. At 37 °C, only the *ssd1-d elp3* and *deg1* mutants, not the *SSD1-v* counterparts, displayed a substantial growth defect. At 39 °C, however, all *ssd1-d* strains, including the wild-type control, were growth-impaired in comparison to their respective *SSD1-v* counterparts (Figure 1A). Of note: the *ssd1-d* allele was previously shown to restrict growth at elevated temperatures [37].

To further test whether the difference in growth phenotypes was due to the allelic variation of *SSD1*, we introduced the *SSD1-v* allele into the *ssd1-d* strains and analyzed phenotypic complementation. Both *ssd1-d* and *ssd1-d deg1* strains were transformed with either an empty vector or a plasmid containing *SSD1-v* ([*SSD1-v*]). As a control, a plasmid carrying *ssd1-d* [*ssd1-d*] was introduced in parallel. As shown in Figure 1B, the thermosensitive growth of wild-type *ssd1-d* and the *deg1* mutant could indeed be suppressed by *SSD1-v*. Growth at elevated temperatures was improved for both the *ssd1-d* and *ssd1-d deg1* strains upon the expression of *SSD1-v* but not with *ssd1-d* or empty vector controls. Thus, the enhanced thermosensitivity of *ssd1-d* strains can be solely attributed to the *ssd1-d* allele, and *SSD1-v* positively affects temperature resistance in the wild type and the tRNA modification mutant. Therefore, the enhancement of *elp3* and *deg1* phenotypes by the *ssd1-d* allele could reflect an additive effect being caused by two independent mechanisms increasing thermosensitivity. Interestingly, the temperature phenotype of an *ssd1-d elp3* mutant was shown to be suppressible by osmotic stabilization [38], but it remained unknown whether this extends to *SSD1-v elp3* or *deg1* mutants. Therefore, we tested the growth of *elp3* and *deg1* in both strain backgrounds at elevated temperatures in the presence of sorbitol (Figure S1). As a result, we observed a mild suppression of temperature phenotypes of the *ssd1-d* strains but not of the *SSD1-v* strains.

**Figure 2:**
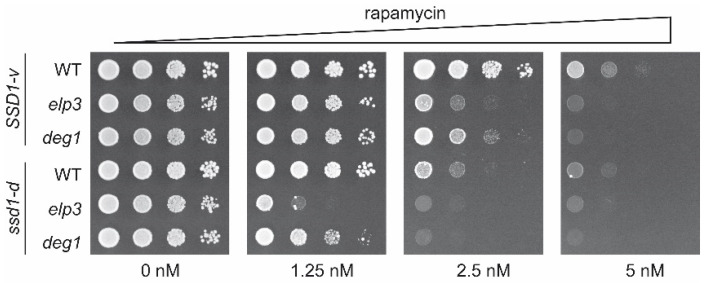
Comparison of the rapamycin sensitivity of tRNA modification mutants in *SSD1-v* and *ssd1-d* backgrounds. WT, *elp3*, and *deg1* mutants of both background strains were serially diluted and spotted on YPD plates containing the indicated amounts of rapamycin. Plates were incubated at 30 °C for 48 h.

In addition to temperature sensitivity, we tested the effect of *SSD1* variation on the rapamycin phenotype of *elp3* and *deg1* mutants. As expected, *deg1* and *elp3* mutants displayed increased rapamycin sensitivity compared to the respective wild-type control [16,30] in both genetic backgrounds (Figure 2). In comparison, however, *elp3* displayed stronger rapamycin sensitivity than *deg1*. Rapamycin sensitivity was generally increased in *ssd1-d* strains. Importantly, the wild-type *ssd1-d* strain also exhibited enhanced sensitivity compared to the *SSD1-v* wild-type strain (Figure 2). To test whether the observed difference in rapamycin sensitivity between the *ssd1-d* and *SSD1-v* strains was due to the loss of *SSD1* function, we analyzed the rapamycin phenotype in an *ssd1* deletion strain and tested whether the *ssd1-d* phenotype could be complemented by the ectopic expression of *SSD1-v*. We found that *ssd1* deletion in the *SSD1-v* strain increased drug sensitivity and *SSD1-v* expression in the *ssd1-d* strain suppressed drug sensitivity (Figure S2). In addition, a complete deletion of *SSD1* in the *SSD1-v* strain background increased temperature and rapamycin sensitivity, both of which were further increased upon the deletion of *DEG1* (Figure S3). Hence, the *SSD1* status itself influences rapamycin resistance. Therefore, increased rapamycin sensitivity in *ssd1-d* tRNA modification mutants may have resulted from an additive effect being caused by independent consequences of tRNA modification loss and Ssd1 defects.

### 2.2. Impact of SSD1 on Genetic Interaction of DEG1 with mcm^5^s^2^U_34_-Relevant Genes

Since *ssd1-d* individually modulated the growth phenotypes of *elp3* and *deg1* tRNA modification mutants, we sought to test the effect of *SSD1* variation on the negative genetic interaction between U_34_ and U_38/39_ modifiers. A strong synergistic growth retardation occurs upon the combination of U_34_ modification defects caused by mutations of either *URM1* or *ELP3* and the deletion of *DEG1* [7]. An *elp3 deg1* double mutant is viable in the *SSD1-v* background but displays a very pronounced growth defect at 30 °C [7]. An *urm1 deg1* double mutant is similarly viable and displays near normal growth at 30 °C but severely delayed growth at 37 °C [7].

**Figure 3:**
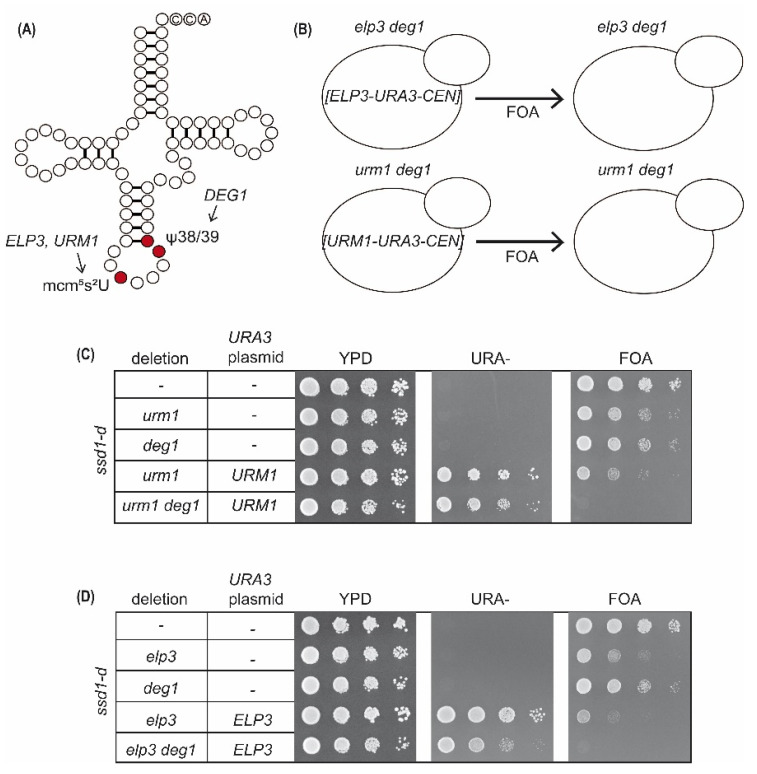
Plasmid shuffle assay to determine genetic interaction between *DEG1* and *ELP3* or *URM1* in the *ssd1-d* strain. (**A**) Scheme indicating position and required genes for mcm^5^s^2^U_34_ and Ψ_38/39_ modifications in tRNA. (**B**) Principle of plasmid shuffle assay involving *elp3 deg1* or *urm1 deg1* double mutants carrying *URA3-CEN* plasmids that provide for *ELP3* or *URM1* wild-type gene functions, respectively. 5-FOA medium (FOA) counterselects against the *URA3*-based plasmids and thus uncovers the double mutant phenotype. (**C**) Result of plasmid shuffle assay in the *deg1 urm1* strain. (**D**) Result of plasmid shuffle assay in the *deg1 elp3* strain. WT and indicated mutants with and without *URA3*-based plasmids were serially diluted and spotted on YPD, URA, and FOA plates. YPD and URA plates were incubated for 48 h, and FOA plates were incubated for 72 h at 30 °C.

To test for the phenotypes of the same double mutants in the *ssd1-d* background, we used the plasmid shuffle approach previously employed to generate the *SSD1-v* double mutant strains. First, *elp3* and *urm1* mutations were each complemented with an appropriate 5-fluoroorotic acid (5-FOA) counterselectable plasmid that provided either *ELP3* or *URM1*. *DEG1* was subsequently deleted, and the ability to lose the *urm1*- and *elp3*-complementing plasmids was monitored by checking growth on 5-FOA-supplemented media (Figure 3A,B). In contrast to the control strains lacking the additional *deg1* deletion, both *elp3 deg1* and *urm1 deg1* double mutants in the *ssd1-d* background were unable to grow on 5-FOA media (Figure 3C,D). Thus, both *elp3 deg1* and *urm1 deg1* double mutants were inviable in the *ssd1-d* strain background. Hence, the observed synthetic sick genetic interaction of *urm1/elp3* and *deg1* in *SSD1-v* was further aggravated in the *ssd1-d* strain, in which a synthetic lethal interaction was observed (Figure 3C,D). This result supports the assumption that both, *elp3* and *deg1* mutations cause more severe phenotypic consequences in the *ssd1-d* strain; consequently, the double mutant is inviable in this background.

Negative growth phenotypes of *SSD1-v deg1 urm1* and *deg1 elp3* double mutant strains were previously shown to be partially suppressible by the overexpression of tRNA^Gl^ ^n^_UUG_ [7]. A functional defect of this tRNA is thought to account for the negative phenotypes of the double mutants, and the higher-than-normal levels of the defective tRNA can likely compensate for functional impairment. To test whether the synthetic lethal interaction between *elp3/urm1* and *deg1* in the *ssd1-d* background can similarly be sup pressed, we repeated the plasmid shuffle approach shown in Figure 3 in the presence of an overexpression construct for tRNA^Gln^_UUG_ or an appropriate empty vector control. As shown in Figure S4, the growth of *ssd1-d elp3 deg1* and *urm1 deg1* strains was rescued in the presence of the tRNA overexpression construct but not the empty vector control. In both double mutants, however, colony formation on 5-FOA media required prolonged incubation times and was thus significantly delayed compared to the wild type or the respective single tRNA modification mutants. Hence, tRNA^Gln^_UUG_ overexpression provides a partial, rather than complete, rescue towards the negative genetic interactions between *ELP3/URM1* and *DEG1* in the *ssd1-d* strain (Figure S4).

### 2.3. Expression of the Gln-Rich Protein Rnq1 in deg1 Mutants

**Figure 4:**
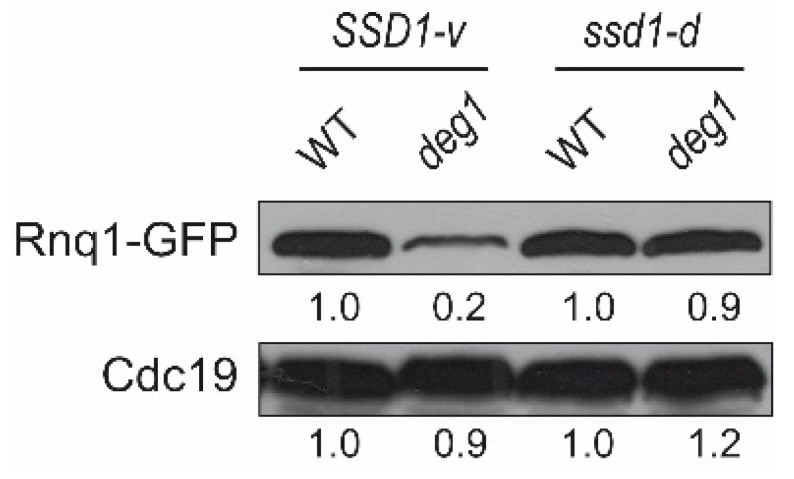
Comparison of protein levels of the glutamine-rich Rnq1-GFP fusion protein in absence of Ψ_38/39_ in *SSD1-v* and *ssd1-d* backgrounds. The total protein extract from indicated strains expressing Rnq1-GFP was used for Western analysis with anti-GFP and anti-Cdc19 antibodies. GFP and Cdc19 signal intensities were normalized to the respective WT intensity.

Multiple lines of evidence indicate a critical dependence of tRNA^Gln^_UUG_-decoding efficiency on Ψ_38_ [4,8,12]. Hence, aggravated phenotypes of *ssd1-d deg1* in comparison to *SSD1-v deg1* could be related to a further elevated decoding defect of tRNA^Gln^_UUG_ in the *ssd1-d* strain. To compare the expression defect related to tRNA^Gln^_UUG_ in the absence of pseudouridylation at position 38/39, we introduced a gene encoding the GFP-tagged Gln-rich prion protein Rnq1 [39] into WT and *deg1* mutants of both *SSD1* backgrounds. Both strains are [*PIN+*], implying the conversion of Rnq1-GFP into amyloid aggregates. Rnq1 contains a high number of Gln codons, and the translation of its mRNA was shown to be reduced in the absence of Ψ_38/39_ [7]. As described before, we observed a clear downregulation of Rnq1-GFP protein levels in the *SSD1-v deg1* mutant in comparison to *SSD1-v*. Surprisingly, however, Rnq1-GFP levels were similar in the *ssd1-d* and *ssd1-d deg1* strains (Figure 4). Thus, the expression of the Gln-rich Rnq1 was found to be improved rather than impaired in the *ssd1-d deg1* mutant compared to the *SSD1-v* strain. Hence, a generally exacerbated translational defect of the Ψ_38_ deficient tRNA appears unlikely to account for the more severe phenotypes observed above in the genetic background of *ssd1-d* cells.

### 2.4. Role of SSD1 Status Variation in Protein Aggregation

Previous work has revealed disturbed protein homeostasis as a major consequence observable in yeast strains lacking mcm^5^s^2^U_34_ modification and/or Ψ_38__/39_ [7,27,29]. The effect is most pronounced in double mutants lacking parts of the mcm^5^s^2^U_34_ modification in combination with the *deg1* mutation or in the complete absence of the mcm^5^s^2^U_34_ modification [7]. The protein homeostasis defect is thought to contribute to mutant growth defects at elevated temperatures since temperature stress-challenges the proteostasis machinery. Given the increased the thermosensitivity of *ssd1-d* and *ssd1-d deg1* strains compared to their *SSD1-v* variants, we investigated potential changes in the content of cellular protein aggregates. We considered the possibility that enhanced growth phenotypes in *ssd1-d deg1* might occur along with enhanced protein aggregation, since a similar effect was observed for an *elp3 ncs2* mutant [30].

**Figure 5:**
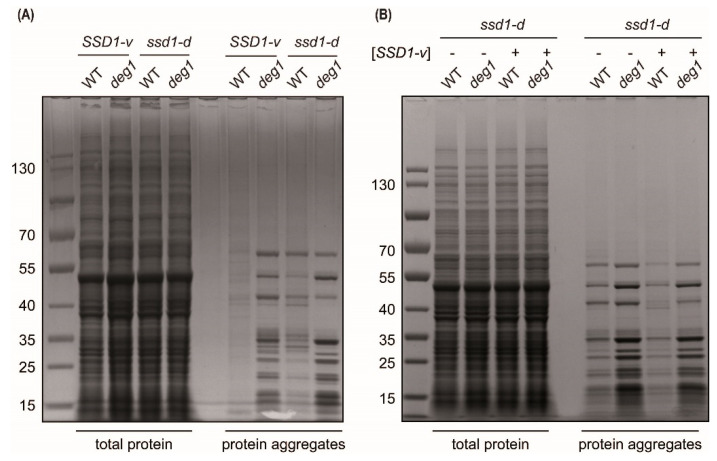
Impact of *deg1* and *ssd1-d* on protein aggregation. Total protein and aggregate contents were extracted from (**A**) WT and *deg1* mutants in both *SSD1-v* and *ssd1-d* backgrounds. (**B**) Total protein and aggregate contents from *ssd1-d* WT and the *ssd1-d deg1* mutant in the presence (+) and absence (−) of plasmid-based *SSD1-v* [*SSD1-v*]. Samples were analyzed by Nu-PAGE and Coomassie staining.

Total protein and protein aggregate contents were extracted from wild-type and *deg1* mutants (*SSD1-v* and *ssd1-d*) and then analyzed on Nu-PAGE gradient gels. As shown in Figure 5A, a *deg1* mutation increased the protein aggregate content in both *SSD1-v* and *ssd1-d* backgrounds. *ssd1-d* displayed slightly more aggregates than *SSD1-v*, and *ssd1-d deg1* showed slightly more aggregates as than *SSD1-v deg1* (Figure 5A and Figure S5).

To test whether the differences in protein aggregation were due to *SSD1* loss of function, we introduced *SSD1-v* plasmids into the *ssd1-d* and *ssd1-d deg1* strains and compared protein aggregation between them. As shown in Figure 5B and Figure S5, the presence of the *SSD1-v* plasmid [*SSD1-v*] reduced the amount of protein aggregates detected in both the *ssd1-d* and *ssd1-d deg1* backgrounds. Thus, *ssd1-d* increased protein aggregation independently of the tRNA modification defect. However, it also increased the tendency of the *deg1* mutant to induce protein aggregates, and this effect might be relevant for the increased temperature sensitivity of *deg1* mutants that we observed in the *ssd1-d* background. Of note, providing *SSD1-v* on a plasmid [*SSD1-v*] suppressed both thermosensitivity and protein aggregation, strongly suggesting a functional correlation.

### 2.5. Chronological Aging in deg1 Mutants

**Figure 6:**
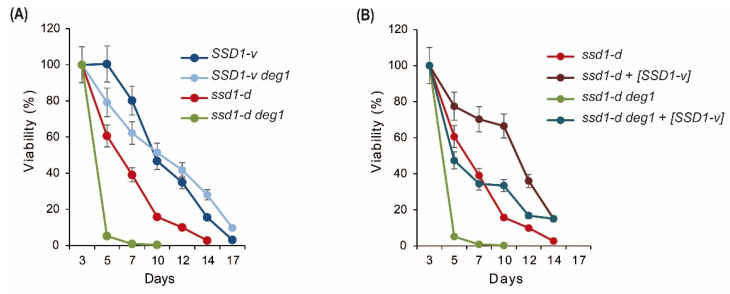
Influence of *SSD1* and *DEG1* on chronological aging. (**A**) Chronological aging was analyzed for the *SSD1-v* WT and the *ssd1-v deg1* mutant in comparison to the *ssd1-d* and *ssd1-d deg1* strains over a time range of 17 days. Viability (%) represents the determined colony forming units (CFU) per ml normalized to the respective value at day 0. The mean of three independent cultures and the standard deviation is given. (**B**) As in (**A**) but with indicated strains in the presence or absence of plasmid-based *SSD1-v* [*SSD1-v*].

In addition to its effect on temperature tolerance, *SSD1* has also been implicated in the process of chronological aging. *ssd1-d* was correlated with a shorter chronological lifespan than *SSD1-v* [36]. Since both *ssd1-d* and *deg1* mutations independently appeared to increase protein aggregation and because this effect might be relevant for long-term stationary phase survival, we tested the effects of *ssd1-d* and *deg1* alone and in combination on chronological aging. A chronological aging assay was performed for the *SSD1-v* and *ssd1-d* strains with and without the *deg1* mutation over a time range of 17 days in the stationary phase (Figure 6A). As expected, the *ssd1-d* strain exhibited a faster decline in viability than the *SSD1-v* strain. At 7 days in the stationary phase, less than 50% viability was retained in the *ssd1-d* cultures, whereas the *SSD1-v* cultures took 10–12 days to reach this point. The *SSD1*-v *deg1* mutant showed a similar decline in viability over time as the wild-type *SSD1-v*, suggesting that in this background, *DEG1* does not represent a major aging factor. However, the *ssd1-d deg1* mutant showed a strongly accelerated loss of viability in comparison to the wild-type *ssd1-d* control (Figure 6A). Hence, in contrast to the *SSD1-v* strain background, the *DEG1* loss-of function appears to contribute to aging in the background of an *ssd1-d* strain.

To test whether the differential effects of *deg1* mutation in the *ssd1-d* and *SSD1-v* strains were exclusively due to the difference in the allelic variant of *SSD1*, we introduced *SSD1-v* plasmids into the wild-type *ssd1-d* and *deg1* mutant and analyzed their chronologic aging. As shown in Figure 6B, the presence of the *SSD1-v* plasmid [*SSD1-v*] improved viability over time in the stationary phase, consistent with the established role of *SSD1* in stationary phase survival. Importantly, however, *ssd1-d deg1* [*SSD1-v*] still displayed an accelerated loss in viability over time compared to the *ssd1-d* [*SSD1-v*] control. Hence, the observation that *DEG1* represents an aging-relevant gene in the *ssd1-d* but not *SSD1-v* strain background is likely due to differences between the two strains other than their none similar *SSD1* locus.

### 2.6. Phenotypic Diversity of Other tRNA Modification Defects in ssd1-d and SSD1-v Strains

**Figure 7:**
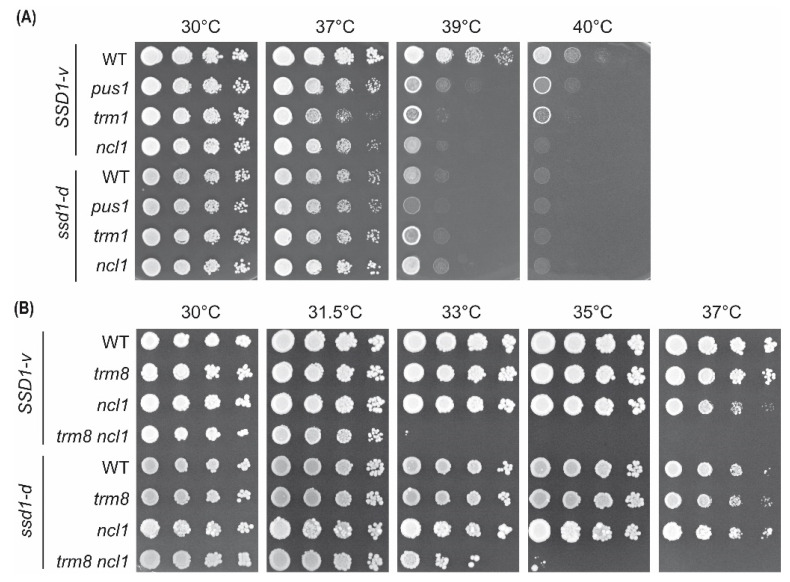
Comparison of the temperature sensitivity of other tRNA modification mutants in *SSD1-v* and *ssd1-d* backgrounds. (**A**) Wild-type (WT) and *pus1, trm1*, and *ncl1* mutants in both *SSD1-v* and *ssd1-d* backgrounds were serially diluted, spotted on YPD plates, and incubated at elevated temperatures for 48 h. (**B**) As in (**A**) but using WT and *trm8*, *ncl1*, and *trm8 ncl1* double mutants in both strain backgrounds.

Besides *elp3* and *deg1*, other tRNA modification genes are linked to the mutant phenotype of retarded growth at elevated temperatures. To test more generally whether temperature-sensitive growth phenotypes of tRNA modification mutants are aggravated by *ssd1-d*, we selected the tRNA pseudouridine synthase Pus1 and the tRNA methyltransferases Trm1 and Ncl1, and we compared their mutant phenotypes in *ssd1-d* and *SSD1-v* backgrounds. In *SSD1-v*, all three mutants (*pus1*, *trm1*, and *ncl1*) displayed robust temperature sensitivity compared to the wild-type *SSD1-v* (Figure 7A), confirming earlier reports [40,41,42]. Interestingly, however, while the *ssd1-d* strain again showed an elevated temperature sensitivity compared to the *SSD1-v* strain, only a modest further enhancement (relative to *ssd1-d*) was seen for *ssd1-d pus1*. Unexpectedly, *ssd1-d ncl1* and *ssd1-d trm1* mutants were not significantly more temperature-sensitive than the *ssd1-d* control (Figure 7A). To further study genetic interaction strength in *ssd1-d* and *SSD1-v* backgrounds, we additionally deleted the *TRM8* methyltransferase gene [43]. In *ncl1 trm8* mutants, a strong negative genetic interaction is well-described and mechanistically linked to the rapid tRNA decay of tRNA^Val^_AAC_ at 37 °C [44]. This results in the complete absence of growth of an *SSD1-v ncl1 trm8* double mutant at 37 °C but not at 30 °C. In the *ssd1-d ncl1 trm8* strain, a robust synthetic temperature sensitivity was observed at 37 °C, but compared to the *SSD1-v ncl1 trm8* mutant, the defect was ameliorated rather than enhanced (Figure 7B).

An additional phenotype described for *SSD1-v trm1* and *pus1* mutants is an enhanced sensitivity against the anticancer drug 5-fluorouracil (5-FU) [40]. A combination of mild heat stress was further shown to strongly increase the efficiency of the drug, potentially by a destabilizing effect on the hypomodified tRNAs. Given the unexpected absence of temperature-sensitive phenotypes for *ssd1-d trm1* and *pus1* mutants, we sought to analyze the 5-FU phenotype in the two strain backgrounds. Compared to the wild-type *SSD1-v*, the *SSD1-v trm1* and *pus1* strains displayed an increased sensitivity to 10 mg/mL 5-FU at 30 °C, but this was not the case for the corresponding *ssd1-d* strains (Figure S6). However, when the drug was applied at 37 °C, both *ssd1-d trm1/pus1* and *SSD1-v trm1/pus1* mutants became strongly sensitized relative to the wild-type controls, thus confirming the expected single mutant phenotypes. Interestingly, the *ssd1-d pus1* and *trm1* mutants appeared to be slightly more resistant to this effect compared to the *SSD1-v* strains (Figure S6). Hence, 5-FU phenotypes of *ssd1-d trm1* and *pus1* in general are confirmed, but they are ameliorated in comparison to the *SSD1-v* strain background.

In conclusion, while *elp3* and *deg1* mutants exhibit more severe growth phenotypes in *ssd1-d* compared to the *SSD1-v* background, this is not generally true for other tRNA modification mutants. In at least three cases (*pus1, trm1,* and *ncl1*), the opposite effect of less severe growth defects in the *ssd1-d* strain can be observed.

## 3. Discussion

In the yeast *S. cerevisiae*, the RNA-binding Ssd1 protein plays prominent roles in cell wall remodeling through the delivery of cell wall protein-encoding mRNA to polarized growth sites [31]. Hence, *ssd1* mutations leading to the loss of Ssd1 function are linked to growth defects at elevated temperatures [37] and in the presence of cell-wall-stress-inducing drugs such as calcofluor white (CFW) [45,46]. More recently, *SSD1* was implicated in the phenotypic variation of *elp3* mutants lacking the catalytic subunit of the tRNA modification complex, Elongator [30]. Pleiotropic *elp3* phenotypes [38,47] are enhanced by the *ssd1-d* allele present in common laboratory strains derived from W303 yeast [30]. *SSD1* is evolutionary conserved in the fungal kingdom and its orthologue in the distantly related fission yeast *S. pombe* similarly encodes a P-body associated RNA-binding protein [48,49]. Thus, the functional conservation of Ssd1 in the fungal kingdom is likely, but phenotypic variation of tRNA modification defects in other fungi remain to be studied.

Mechanistically, a functional Elongator complex is required to maintain the translational capacity of tRNA^Lys^_UUU_ and tRNA^Gln^_UUG_ [47,50,51,52]. Deg1 was also shown to be required for the functionality of tRNA^Gln^_UUG_, and the strong negative genetic interactions of *DEG1* with Elongator-related genes are due to an additive functional impairment of this tRNA [7,8,16,29]. However, prior to this study, it remained unclear whether *DEG1* also shares the recently described negative genetic *SSD1* interaction with *ELP3* [30].

In this study, we demonstrated that this is the case, as growth phenotypes of both *elp3* and *deg1* were found to be more pronounced in the W303 *ssd1-d* strain compared to the BY4741 *SSD1-v* strain, including sensitivity to elevated temperatures and the TORC1 inhibitor drug rapamycin. Additionally, the genetic interactions between *ELP3*, *DEG1*, and *URM1* were found to be enhanced from synthetic sickness in the *SSD1-v* strain [7] to synthetic lethality in the *ssd1-d* strain (Figure 3). To exclude the option that the observed variation in stress phenotypes was due to differences other than the *SSD1* locus, we verified that (i) growth phenotypes in the *ssd1-d* strain were rescued by the ectopic expression of *SSD1-v* (Figure 1B) and that (ii) growth phenotypes in the *SSD1-v* strain were similarly enhanced by the complete loss of *SSD1* (Figure S3). Phenotype assessment, however, clearly demonstrated that *ssd1* mutation itself is linked to the same phenotypes as for *elp3* and *deg1* (Figure 1A and Figure S3). Hence, it appears that the observed phenotypic variation of *elp3* and *deg1* mutants by *ssd1* is caused by related cellular effects of either mutation alone rather than a tRNA-modification-specific effect of *ssd1*. Our observation that *deg1* phenotypes are enhanced by *ssd1-d* (Figure 1) but not the expression defect of gene encoding a Gln-rich protein (Figure 4) further supports our assumption that *ssd1-d* does not specifically aggravate the effect of modification loss at the level of translation. It currently remains unknown why the relative reduction of Rnq1-GFP signal strength is actually reduced in the *ssd1-d deg1* mutant. An impact of the strain background on the amyloid aggregate formation potential of the protein might contribute to the observed differences.

Both Deg1 and Elongator have been implicated in the maintenance of protein homeostasis [7,28,29,53,54]. A deficiency in protein homeostasis is thought to account for stress phenotypes of certain combined mutants involving Elongator, tRNA thiolation, and/or Deg1 defects (e.g., temperature sensitivity) [7,28]. Since Ssd1 also has a documented role in protein disaggregation by influencing the ability of heat shock protein Hsp104 to bind protein aggregates [55], the phenotypic variation of *deg1* and *elp3* could be at least partly mediated by effects on protein aggregation. Indeed, protein homeostasis defects of a combined *elp3 ncs2* mutant [30,56] and of a *deg1* single mutant (Figure 5) were moderately elevated by the presence of the *ssd1-d* allele. It is worth noting that the presence of the *ssd1-d* allele alone already increased the amount of detectable cellular protein aggregates (Figure 5A). Thus, Ssd1 could prevent protein aggregation in a mechanistically distinct way compared to the tRNA modifications, potentially involving the described effect on Hsp104 function. Such an independent effect on protein homeostasis could explain the observed additive phenotypes of *ssd1-d* in conjunction with *elp3* or *deg1* mutations.

In addition to the effect on protein homeostasis, a phenotypic similarity between *elp3*, *deg1*, and *ssd1* could also be related to similar effects of either mutation on cell wall integrity. Like *ssd1* mutant cells, *elp3* mutants display an increased sensitivity to cell wall stressor CFW [38], and combined mutants involving *elp3*, *uba4*, *urm1*, and *deg1* mutations in the *SSD1-v* background were shown to exhibit hyperpolarized growth and cell lysis phenotypes that likely imply cell wall defects [7,27]. Along these lines, it was further demonstrated that *elp3* mutant phenotypes are partially suppressed by genetically upregulating the cell wall integrity (CWI) pathway [30] and by osmotic stabilization [38]. Similar to *ssd1-d elp3*, we found that *ssd1-d deg1* mutants are also in part phenotypically suppressed by osmotic stabilization (Figure S1). Hence, if *ssd1* and *elp3/deg1* individually induced cell wall defects by distinct mechanisms, these could be elevated in their respective double mutants with a single tRNA modification defect and a null or truncated allele of *SSD1*. In support of this idea, we found that like *elp3*, the *deg1* mutation also increased CFW sensitivity and a double *ssd1 deg1* mutant showed additivity, in not only rapamycin and temperature sensitivity but also CFW sensitivity (Figure S3). While the molecular basis for rapamycin sensitivity of different tRNA modification mutants is not fully understood, it is noteworthy that modification mutants involving *elp3* and/or *deg1* mutations display hallmarks of reduced TORC1 activity [29,57]. Since TORC1 has, amongst others, a role in the maintenance of cell wall integrity [58,59], it could be hypothesized that rapamycin increases the chronic cell wall integrity defects of the tRNA modification mutants, which are further elevated upon the loss of Ssd1 and result in additive phenotypes in combination with *ssd1-d*.

We additionally observed that the *deg1* mutation is linked to reduced chronological lifespan, which was specifically obvious in the *ssd1-d* strain background (Figure 6). It was previously demonstrated that *SSD1* mutations affect the transcript levels of longevity genes and thereby drastically reduce chronological lifespan [36] Additionally, proper CWI signaling positively contributes to long-term survival of the stationary phase [60]. Hence, additive effects of *deg1* and *ssd1* mutations on the CWI and mRNA levels of longevity genes might explain the *ssd1-d*-specific aging phenotype of *deg1*. Additionally, the clearance of protein aggregates that accumulate during chronological aging [61] might influence long-term stationary phase survival. Therefore, the shortened lifespan of the *ssd1-d deg1* mutant might partly be related to the elevated protein aggregation that we detected (Figure 5). However, since a W303 *deg1 ssd1-d* [*SSD1-v*] mutant showed a reduced chronological lifespan compared to the *ssd1-d* [*SSD1-v*] control (Figure 6B), other genetic differences and factors between BY4741 and W303 probably contribute to the short-lived phenotype observed in W303 *deg1* rather than BY4741 *deg1* cells.

Unexpectedly, other tRNA modification defects that share the temperature-sensitive phenotype with *elp3* and *deg1* did not generally exhibit enhanced phenotypes in the *ssd1-d* W303 strain compared to *SSD1-v* BY4741. On the contrary, we observed ameliorated effects for *pus1, trm1*, and *ncl1* in W303. This was not limited to temperature sensitivity, as it also extended to sensitivity against the anticancer drug 5-FU, which was weakened rather than enhanced in W303 *pus1* and *trm1* mutants compared to the BY4741 counterparts (Figure S6). Intriguingly, phenotypic enhancement by *ssd1-d* appears to be refined to two specific modification defects that induce a pleiotropic set of phenotypes overlapping with *ssd1*. Of particular relevance could be the CFW sensitivity indicative of cell wall defects and shared between *ssd1*, *elp3*, and *deg1* but not, for instance, with other pseudouridine synthase defects (Figure S3B). Hence, the phenotypic variation of tRNA modification defects by *ssd1* could be restricted to those tRNA modifications that play roles in cell wall integrity and might therefore reflect additive cell wall damage.

## 4. Conclusions

We demonstrated that in *S. cerevisiae*, the RNA-binding Ssd1 protein influences the cellular consequences of tRNA modification loss. In addition to the previously described *SSD1-*dependent phenotypic modulation of *elp3* mutants lacking specific wobble uridine modifications, a similar effect was observable in *deg1* mutants defective in U38 and U39 pseudouridylation. However, phenotypes associated with other tRNA modification defects were not similarly affected. Genetic evidence suggests that *elp3*, *deg1*, and *ssd1* mutants share cell wall integrity defects that might be responsible for additive negative phenotypes.

## 5. Materials and Methods

### 5.1. Strains and General Methods

The strains of *Saccharomyces cerevisiae* used in this study are listed in Supplementary Table S1, and standard methods were used for yeast growth and maintenance [62]. When plasmid maintenance was required, a synthetic complete medium lacking either leucine or uracil (depending on the selectable marker of the plasmid) was used. Genomic deletions were generated with the help of PCR [63] and oligonucleotides targeting *ELP3*, *URM1*, *DEG1*, *PUS1*, *TRM1*, *TRM8*, or *NCL1* (Supplementary Table S2). Replacements were confirmed by PCR using primers located outside of the target genes (Supplementary Table S2). For temperature and drug sensitivity assays, freshly grown yeast cells were resuspended in sterile water and adjusted to an initial optical density (OD_600nm_) of 1. These suspensions were used for 10-fold serial dilutions and spotted on YPD plates with or without the presence of indicated drugs. For temperature assays, identical replicate plates were prepared from the same cell dilutions and incubated at different temperatures.

### 5.2. Plasmid Construction and Shuffling

For the deletion of *DEG1* in *urm1:SpHIS3* or *elp3::SpHIS3* strains, they were first transformed with pFF8 (*ELP3*; [27]) or pHA-URM1 (*URM1*; [64]) to genetically complement the genomic deletions. Subsequently, *DEG1* was deleted by using a PCR-generated deletion cassette (*deg1:SpHIS5*), and the *URA3* plasmids pFF8 or pHA-URM1 were eliminated by growth on minimal media containing uracil and 5-fluoro-orotate (1 mg/mL). For the plasmid-based complementation of *ssd1-d*, the *SSD1-v* plasmids pPL091 (*LEU2*) and pPL092 (*URA3*) [58] were used. For control purposes, the *ssd1-d* plasmid pPL093 (*URA3*) [58] was utilized. tRNA^Gln^_UUG_ overexpression used pRK55 [7]. Rnq1 was expressed as a GFP fusion from plasmid p1332 [65].

### 5.3. Protein Isolation and Western Blotting

Protein extracts were obtained from cells grown to OD_600nm_ = 1 using disruption with glass beads [66]. Protein yield was quantified according to [67], and equal amounts of total protein (50 µg) from different strains were used for loading the gels. Transfer and detection were done as described previously [38] and involved anti-GFP (Santa Cruz Biotechnology, Dallas, TX, USA) and anti-Cdc19 antibodies.

### 5.4. Isolation of Protein Aggregates

Aggregated proteins were obtained from 50 mL YPD cultures grown to OD_600nm_ = 1, as previously described [28,68]. This method is based on the isolation of aggregated proteins from total protein extracts by centrifugation. Identical amounts of total protein extract were subjected to centrifugation and washing steps as previously described [27,65]. The obtained aggregate pellet was dissolved and analyzed by denaturing SDS acrylamide gel electrophoresis and Coomassie staining. To control for the identical input of total protein, a portion of each extract (25 µg) was analyzed along with the aggregate samples. After breaking cells by sonification, a Bradford assay was used to determine the obtained amount of protein for each strain; 4 mg of total protein were subjected to centrifugation and washing [68]. Aggregates from the pellet were boiled in a Laemmli buffer and were separated on NuPAGE Bis–Tris 4–12% gradient gels. As a control, 25 µg of the total protein extract used for aggregate isolation were run on the same gel.

### 5.5. Chronological Aging Assay

Yeast chronological lifespan (CLS) was determined according to [69]. Freshly streaked colonies were inoculated into a preculture consisting of a 2 mL synthetic complete dextrose (SDC) medium and incubated at 30 °C overnight. Main cultures were inoculated at OD_600nm_ = 0.1 in a 10 mL SDC medium in Erlenmeyer flasks covered with aluminum foil. The optical density of the main culture was measured until the stationary phase, considered as time point day zero with an initial survival of 100%, was reached [69]. To determine viability, cells from each flask were diluted and plated on two YPD plates that were incubated at 30 °C until colonies appeared. Colony counts were used to calculate colony forming units per ml (CFU/mL) for each culture and time point (0–17 days). Relative viability represents the CFU/mL value normalized to the value obtained for day zero. Each strain was analyzed using three independent cultures that were cultivated in parallel.

## Data Availability

The data underlying this article will be shared on reasonable request from the corresponding author.

## Supplementary Materials

The following are available online at https://www.mdpi.com/article/10.3390/ijms22168753/s1, Table S1: Strains used or generated in this study. Table S2: Oligonucleotides used in this study. Figure S1: Temperature and drug sensitivity of *elp3* and *deg1* modification mutants in both *SSD1* backgrounds. Figure S2: Rapamycin sensitivity in the presence and absence of *SSD1.* Figure S3: Drug sensitivities of gene deletion mutants in the *SSD1-v* background. Figure S4: Rescue of the lethality of *deg1 urm1* and *deg1 elp3* strains. Figure S5: Densiometric analysis of protein aggregation shown in Figure 5. Figure S6: 5-fluorouracil (5-FU) phenotype of tRNA modification mutants in *ssd1-d* and *SSD1-v* backgrounds.
