# Supplementary material for: Role of SSD1 in Phenotypic Variation of Saccharomyces cerevisiae Strains Lacking DEG1-Dependent Pseudouridylation
Source: Int J Mol Sci. 2021 Aug 15;22(16):8753. doi: 10.3390/ijms22168753 (PMC8396022; doi:10.3390/ijms22168753)
Supplement: Supplementary file 1 [file ijms-22-08753-s001.zip › ijms-1336245-supplementary.pdf]

International Journal of
Molecular Sciences

MDPI

Supplementary Materials

Role of *SSD1* in phenotypic variation of *Saccharomyces cerevisiae* strains lacking *DEG1* dependent pseudouridylation

Bahar Khonsari[1], Roland Klassen [1,*] and Raffael Schaffrath[1,*]

[1] Affiliation 1; Institut für Biologie, Fachgebiet Mikrobiologie, Universität Kassel, Heinrich-Plett-Str. 40, D-34132 Kassel, Germany

* Correspondence: roland.klassen@uni-kassel.de (R.K.); schaffrath@uni-kassel.de (R.S.)

Table S1. Strains used or generated in this study.

Strain	Genotype	Reference/source
S. cerevisiae BY4741	*MATa, his3Δ, leu2Δ, met15Δ, ura3Δ*	Euroscarf, Frankfurt
S. cerevisiae elp3	BY4741 *elp3Δ::KanMX4*	Euroscarf, Frankfurt
S. cerevisiae urm1	BY4741 *urm1Δ::KanMX4*	Euroscarf, Frankfurt
S. cerevisiae deg1	BY4741 *deg1Δ::KanMX4*	Euroscarf, Frankfurt
S. cerevisiae pus1	BY4741 *pus1Δ::KanMX4*	Euroscarf, Frankfurt
S. cerevisiae pus4	BY4741 *pus4Δ::KanMX4*	Euroscarf, Frankfurt
S. cerevisiae pus6	BY4741 *pus6Δ::KanMX4*	Euroscarf, Frankfurt
S. cerevisiae pus7	BY4741 *pus7Δ::KanMX4*	Euroscarf, Frankfurt
S. cerevisiae trm1	BY4741 *trm1Δ::KanMX4*	Euroscarf, Frankfurt
S. cerevisiae trm8	BY4741 *trm8Δ::KanMX4*	Euroscarf, Frankfurt
S. cerevisiae ncl1	BY4741 *ncl1Δ::KanMX4*	Euroscarf, Frankfurt
S. cerevisiae ssd1	BY4741 *ssd1Δ::KanMX4*	Euroscarf, Frankfurt
S. cerevisiae W303-1B	*MATα {leu2-3,112 trp1-1 can1-100 ura3-1 ade2-1 his3-11,15}*	[33]
S. cerevisiae elp3	W303-1B *elp3Δ::SpHIS*	this work
S. cerevisiae urm1	W303-1B *urm1Δ::SpHIS*	this work
S. cerevisiae deg1	W303-1B *deg1Δ::SpHIS*	this work
S. cerevisiae pus1	W303-1B *pus1Δ::SpHIS*	this work
S. cerevisiae trm1	W303-1B *trm1Δ::SpHIS*	this work
S. cerevisiae trm8	W303-1B *trm8Δ::SpHIS*	this work
S. cerevisiae ncl1	W303-1B *ncl1Δ::SpHIS*	this work

Table S2. Oligonucleotides used in this study.

Oligonucleotide	Sequence	Target
koELP3fw	AGTCCTAAAAGCACCTAAGGAAAATCGAAGAACA<br>CCCTGACAAAGCAGCTGAAGCTTCGTACGC	pUG27/*ELP3*
koELP3rv	AAAACCGGCCATGTCGGCGGCACATAAAAGTTC<br>TATTTACCTTTAGCATAGGCCACTAGTGGATCTG	pUG27/*ELP3*
Elp3fw	CGATAAGACAGTGAGAGAAGG	*ELP3*
Elp3rv	AACACATGCAGCAGTTACTCC	*ELP3*
koURM1fw	CAATACTGATTTCTGATACTAAAACGAGATAGGT<br>TAATAGCAAAATCGGGCAGCTGAAGCTTCGTAC GC	pUG27/*URM1*
koURM1rv	CTTTATATATATATATATGTAGCTGCTTCTTAAAAAT<br>TATTTGCTGCTATTTGCATAGGCCACTAGTGGAT CTG	pUG27/*URM1*
urm1fw	CACATACCGGATTATGTTCCTTCCC	*URM1*
urm1rv	ACTGGAATGGTAGAGGTCTTTTGGG	*URM1*

koDeg1fw	GGTGCCCACATGCAATCTTTACTGCCCTACTATA ACCTCCCTTGACAGCTGAAGCTTCGTACGC	pUG27/*DEG1*
koDeg1rv	GAAATATAGTCTTCAAGGTTATATTATACAGGTTT ATATATTATTGCATAGGCCACTAGTGGATCTG	pUG27/*DEG1*
Deg1fw	TGGGCTCAGCTCATCTTG	*DEG1*
Deg1rv	GTGTCGGTCGTCCAATATC	*DEG1*
koPUS1fw	ATAAAGGACAATAAAGTGCTAGTAAATAACAATTATAAGTGATATCAA GGCAGCTGAAGCTTCGTACGC	pUG27/*PUS1*
koPUS1rv	ATGTCAATGCCTTAGAAATTAAAGTTGGTAAGAAAGAAGGAAAGGGCA ACGCATAGGCCACTAGTGGATCTG	pUG27/*PUS1*
pus1fw	GATGCGGGTAACTATTAGCC	*PUS1*
pus1rv	GCGCAATGAGCTTTCCAAGG	*PUS1*
koTRM1fw	ACAGATCCTGAGCAGTCATAAGTTGATACCTTTCCTCTTACAATGTAGAT CAGCTGAAGCTTCGTACGC	pUG27/*TRM1*
koTRM1rw	GGGCGGATCCTTAGTTTCTTACGTTTTAGCTCTAACACTAATCAAAATTC GCATAGGCCACTAGTGGATCTG	pUG27/ *TRM1*
trm1fw	TCTGCTATTGTGCCGCTATG	*TRM1*
trm1rv	CATACATACTGCCCTCCTG	*TRM1*
koTRM8fw	TAGATTCAGCAGTTCCCATAGGATAAAATTTTCAAGCGTTTATTGTTAAG CAGCTGAAGCTTCGTACGC	pUG27/*TRM8*
koTRM8rw	TAAGAAATAGTTATGTATATGTGGTAAATTGTTCTAGTTATACATCTATG GCATAGGCCACTAGTGGATCTG	pUG27/ *TRM8*
Trm8fw	GGATGTGGAGTCAACTACAG	*TRM8*
Trm8rv	TGCGGCTCAAATAACCTGGC	*TRM8*
koNCL1fw	TCTAACACTTCCTTTTATCTACACTGTAATCCGAAGAATACACTATAAGG CAGCTGAAGCTTCGTACGC	pUG27/*NCL1*
koNCL1rv	AATAATATACATTTACTTTACAGTGGAGGGGATAAGAAACATGATAACT AGCATAGGCCACTAGTGGATCTG	pUG27/ *NCL1*
ncl1fw	CGCTGAGTTCTTCCAAAGAC	*NCL1*
ncl1rv	CCAACTCCGCAGGTCTTTCG	*NCL1*

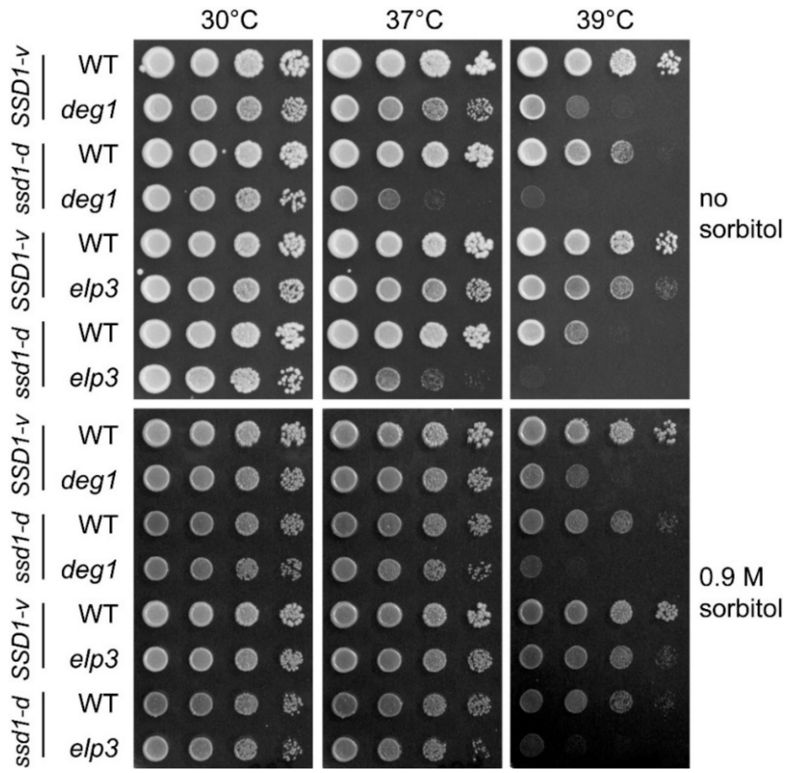

Figure S1. Suppression of temperature sensitivity of *elp3* and *deg1* mutants by osmotic stabilization. Indicated strains in *SSD1-v* and *ssd1-d* backgrounds were serially diluted and spotted on YPD plates without and with 0.9 M sorbitol. Plates were incubated at the indicated temperatures for 48 h.

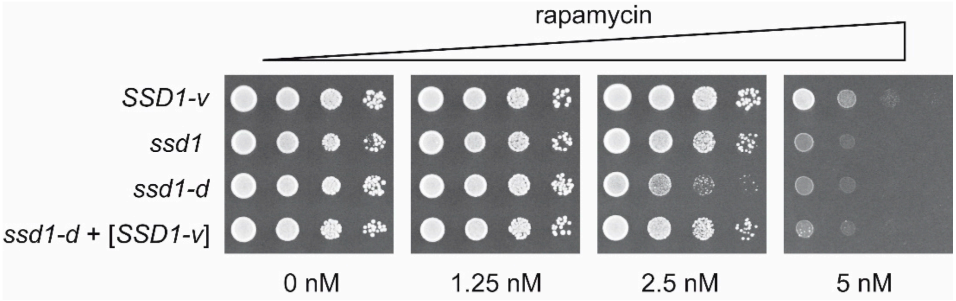

Figure S2. Rapamycin sensitivity in presence and absence of *SSD1.* Yeast strain backgrounds: *SSD1-v* (BY4741); *ssd1* (BY4741); *ssd1-d* (W303-1B); *ssd1-d* + [*SSD1-v*] (W303-1B with *SSD1-v* plasmid pPL092). Strains were spotted on YPD plates with the indicated concentrations of rapamycin and incubated for 48 h at 30 °C.

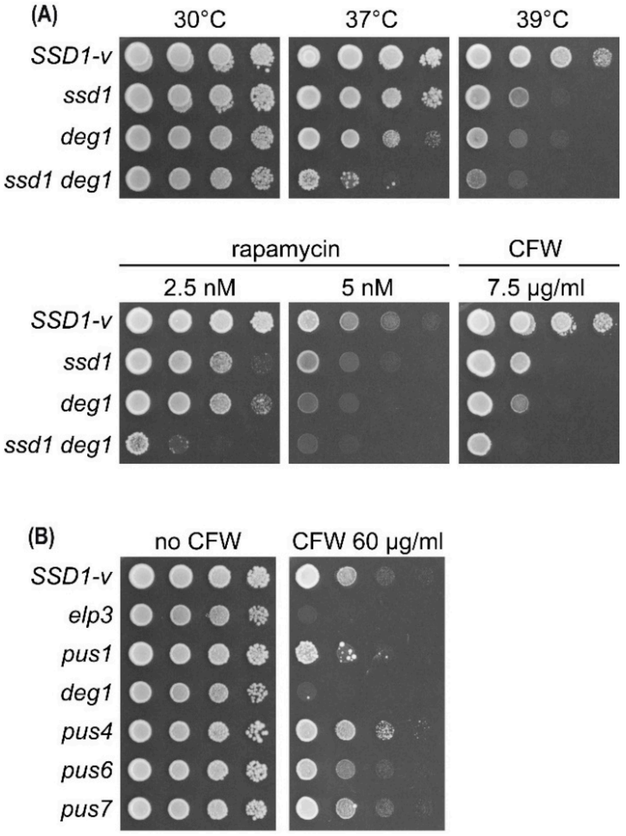

Figure S3. Drug sensitivities of gene deletion mutants in the *SSD1-v* background. (**A**) *SSD1-v* wild type, *ssd1* and *deg1* deletion mutants were serially diluted and spotted on YPD plates containing no drug or the indicated amounts of rapamycin or calcofluor white (CFW). Drug free plates were incubated at the indicated temperatures and rapamycin or CFW plates were incubated at 30 °C for 48 h (**B**) *SSD1-v* wild type and indicated deletion mutants were serially diluted and spotted on YPD plates without or with 60 µg/mL CFW and incubated at 30 °C for 48 h. All strains are in the BY4741 background.

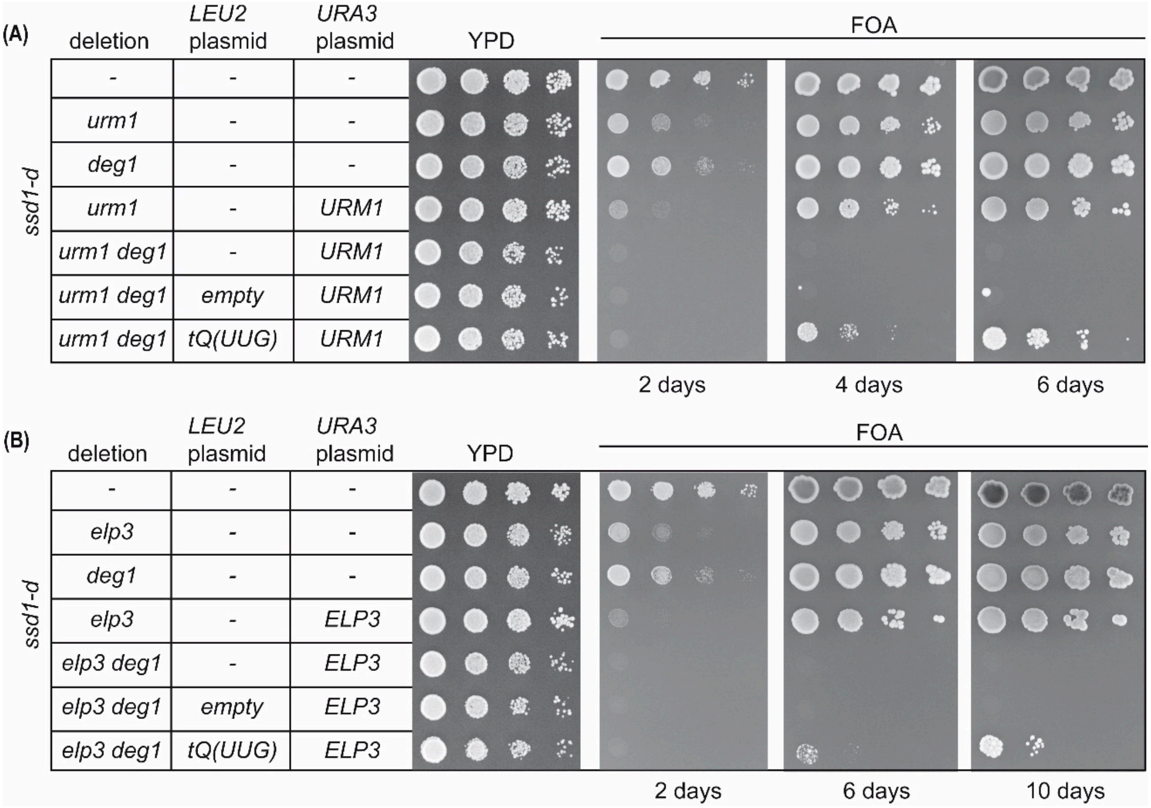

Figure S4. tRNA overexpression rescue of the synthetic lethality of combined tRNA modification mutants. All strains are in the W303-1B background and contain indicated gene deletions and in addition either no plasmid (-), empty *LEU2* vector pRS425 (empty), *LEU2*-tRNA$^{Gln}{}_{UUG}$ high copy (h.c.) vector pRK55 (*tQ(UUG)*) or single copy (s.c.) *URA3* vectors providing *ELP3* and *URM1*, respectively. (**A**) tRNA overexpression rescue of *urm1 deg1*. (**B**) tRNA overexpression rescue of *elp3 deg1*. Strains were serially diluted and spotted on YPD and FOA plates. YPD plates were incubated for 48 h and the FOA plates were incubated as indicated at 30 °C.

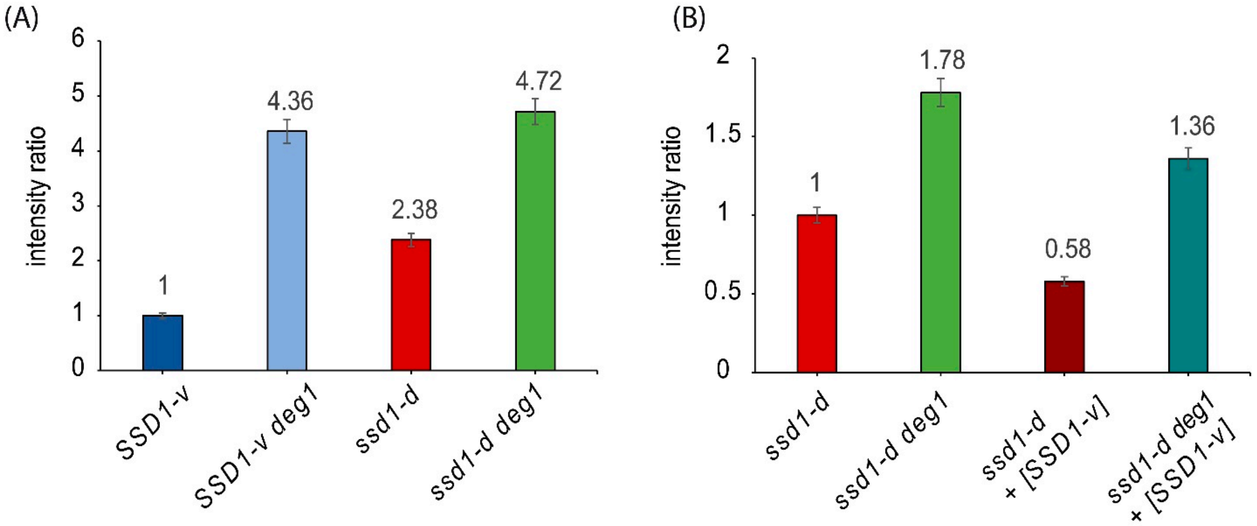

Figure S5. Densiometric analysis of protein aggregation shown in Figure 5. (**A**) Quantification of aggregate band intensity in Figure 5A (**B**) Quantification of aggregate band intensity in Figure 5B. Major aggregate bands were quantified using ImageJ and average intensity normalized to average intensity of signals in *SSD1-v* (A) or *ssd1-d* (B).

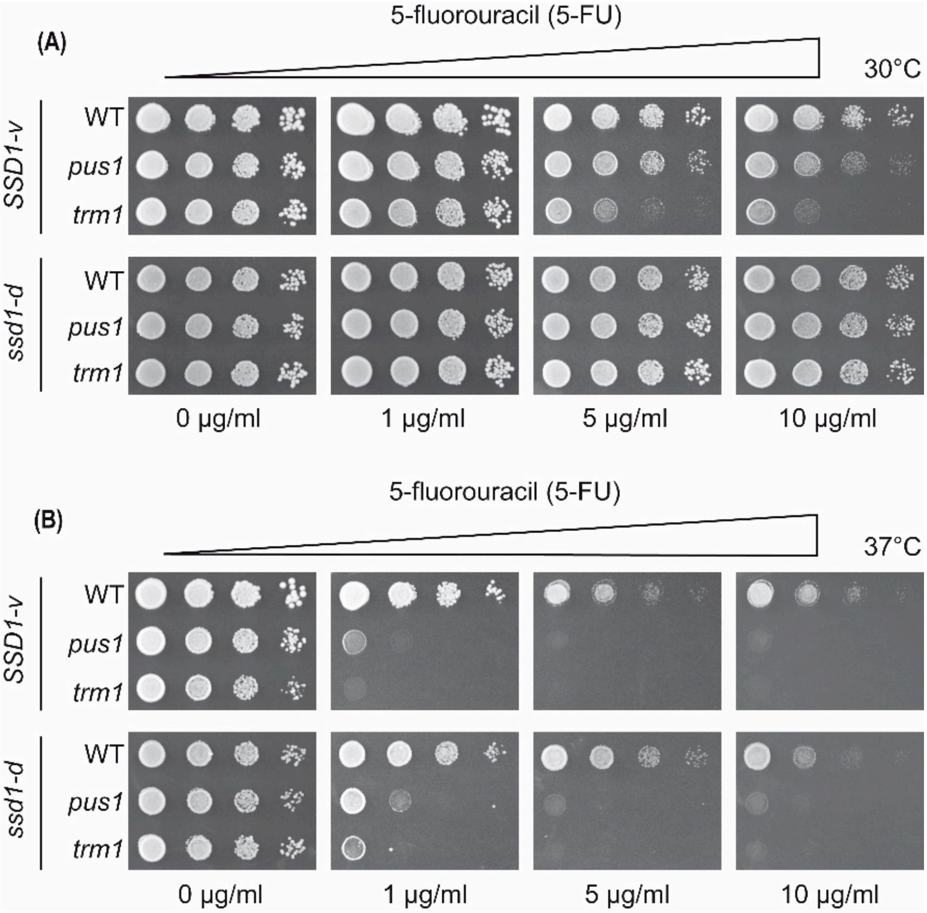

Figure S6. 5-fluorouracil (5-FU) phenotype of tRNA modification mutants in *ssd1-d* and *SSD1-v* backgrounds. (**A**) WT, *pus1* and *trm1* mutants in both *SSD1* backgrounds were spotted on YPD plates containing the indicated amounts of 5-FU and incubated at 30 °C for 48 h. (**B**) Identical cell suspensions as in (**A**) were spotted on YPD plates containing the indicated amounts of 5-FU and incubated at 37 °C for 48 h.
